# Commentary: Locus Coeruleus Ablation Exacerbates Cognitive Deficits, Neuropathology, and Lethality in P301S Tau Transgenic Mice

**DOI:** 10.3389/fnins.2018.00401

**Published:** 2018-06-06

**Authors:** Matthew J. Betts, Alexander J. Ehrenberg, Dorothea Hämmerer, Emrah Düzel

**Affiliations:** ^1^German Center for Neurodegenerative Diseases (DZNE), Magdeburg, Germany; ^2^Institute of Cognitive Neurology and Dementia Research, Otto von Guericke University Magdeburg, Magdeburg, Germany; ^3^Memory and Aging Center, Weill Institute for Neurosciences, University of California, San Francisco, San Francisco, CA, United States; ^4^Institute of Cognitive Neuroscience, University College London, London, United Kingdom; ^5^The Wellcome Trust Centre for Neuroimaging, University College London, London, United Kingdom

**Keywords:** locus coeruleus, norepinephrine, hippocampus, tauopathy, learning and memory, neuropathology

Alzheimer's disease (AD) is characterized by progressive neuron loss as well as the accumulation of neurofibrillary tangles, otherwise referred to as tau pathology, and β-amyloid plaques. Second to neuron loss, tau pathology is the best predictor of cognitive decline in AD (Giannakopoulos et al., 2003). Recently, tau pathology onset in the locus coeruleus (LC) has been shown to consistently precede cortical tau pathology and increase in severity along Braak staging (Braak et al., 2011; Stratmann et al., 2016; Ehrenberg et al., 2017).

The LC is a group of noradrenergic neurons in the pons found in nearly every vertebrate lineage with vast projections, via the dorsal tegmental bundle, to regions in the cerebellum, mesencephalon, diencephalon, and telencephalon, as well as the spinal cord. In humans the LC is subject to excessive metabolic demand, which may render itself a vulnerable target for pathology (Sharma et al., 2010). In large postmortem samples, the burden of hyperphosphorylated tau in the LC significantly increases alongside signficant decreases in LC volume at the earliest stage of AD pathology (Ehrenberg et al., 2017; Theofilas et al., 2017) which may lead to the propogation of tau pathology into noradrenergic projecting cortical regions. Additionally, there is evidence that early degeneration of the LC may contribute to global pathology in AD due to norepinepherine (NE) dysfunction. In animal models, LC ablation has been shown to exacerbate β-amyloid plaque deposition and neuroinflammation, as well as cognitive decline (Heneka et al., 2006; Kalinin et al., 2007; Jardanhazi-Kurutz et al., 2010; Rey et al., 2012). However, it has remained unclear how LC ablation may influence tau pathology or to what extent LC neurodegeneration and tau pathology may interact to impact cognitive dysfunction in AD. In a recent issue of *The Journal of Neuroscience*, Chalermpalanupap et al. (2018) investigated the effects of LC ablation on tau pathology to further illustrate the implications of NE dysregulation on neuropathological hallmarks of AD.

Chalermpalanupap et al. utilized a selective neurotoxin, DSP-4, that targets noradrenergic cells of the LC, to investigate the effects of LC ablation longitudinally across three time points in a mouse-model of tauopathy (P301S tau) in comparison to wild-type and non-LC-ablated P301S mice. Behavioral examination revealed that LC ablation significantly exacerbated cognitive impairment in P301S mice evident by a significant decrease in hippocampal-dependent contextual, but not cued fear memory responses. Interestingly a signficant decrease in contextual fear learning was only detected in the DSP-4 treated P301S mice suggesting that tau pathology and LC degeneration act synergistically to impair learning and memory. Upon histological examination of the hippocampal formation, increased neuron loss was detected in the LC-ablated compared to non-LC-ablated P301S mice, yet significantly higher levels of hyperphosphorylated tau in the LC-ablated P301S mice were only detected at the second of the three assessed time points. This may indicate that dysregulation of NE exacerbates the physiologic and behavioral response to tau, with only modest increases in the propagation of tau pathology. Furthermore this may also suggest that the NE system may attempt to compensate for the ensuing pathology by upregulating noradrenergic receptors and/or increasing activity of remaining noradrenergic neurons as reported during human AD progression (Herrmann et al., 2004).

The results presented by Chalermpalanupap et al. (2018) importantly indicate that LC neurodegeneration in the presence of existing tau pathology is not only a consequence of AD, but also drives associated clinical and pathological manifestations. Interestingly, DSP-4-induced changes were accompanied by only a modest increase in tau pathology despite the severe functional deficits, suggesting that increased tau pathology cannot fully account for the global effects of LC degeneration. Previous studies demonstrating that LC lesions exacerbate ß-amyloid plaque deposition and cognitive decline using amyloid models have all shown that neuroinflammation plays a role in mediating these effects. In line with this, Chalermpalanupap et al. similarly demonstrated that an increase in activated microglia and astrocytes in the hippocampus of lesioned P301S mice was higher than that observed by the non-lesioned P301S mice. Thus, increased inflammation may have also contributed to the accelerated loss of hippocampal neurons and cognitive decline following LC ablation in the P301S mice. Furthermore the influence of LC degeneration on tau pathology may also relate to a direct loss of NE, or indirectly via loss of additional LC neuromodulators such as brain-derived neurotrophic factor or galanin. Interestingly the same group recently reported that chemogenetic activation of the LC rescues hippocampal-dependent learning and memory in a rat model of AD with both tau and ß-amyloid pathology, which may suggest that the acute neuromodulatory effects of NE may be more important than a chronic, neurotrophic influence on pathology (Rorabaugh et al., 2017). However, at present, the exact mechanisms underlying the interaction between LC degeneration and tau remain to be determined. Further studies directly targeting LC neurons coupled with pharmacological approaches disrupting NE-mediated neurotransmission will be important to elucidate how LC neurodegeneration may influence tau pathology.

It is unclear from the study to what extent the entorhinal cortex (EC), a region that also receives dense projections from the LC, was also affected in P301S mice. This relationship would be important to ascertain, since in humans it would be expected that tau would appear in the transentorhinal and entorhinal cortex prior to the hippocampus (Braak and Braak, 1991). However, the EC may not be a focal point in this animal model since P301S mutant tau has a different seeding capacity compared to tau aggregates found in human AD, and thus may spread differently (Woerman et al., 2017). Moreover mutant tau in genetically modified animals such as P301S mice may have different susceptibilities to post-translational modifications found in human tauopathies and should be considered when interpreting these findings.

In humans, postmortem studies have revealed significant correlations between LC cell death and decreased cortical NE levels with severity and duration of dementia in AD (Yates et al., 1983; Kelly et al., 2017), and that the neural density of the LC-NE nuclei may prevent cognitive decline (Wilson et al., 2013). Thus, understanding the role of LC degeneration in AD pathogenesis and related changes in NE modulation may provide important insights into the underlying mechanisms of the disease and improve earlier detection of disease pathology. Recent advances permit monitoring the integrity of the LC-NE system *in vivo*, i.e., using novel MRI techniques to probe changes in LC structure (Betts et al., 2017; Priovoulos et al., 2017) which may be used in tandem with pupillometry to assess LC function (Hämmerer et al., 2018). It is hoped that such approaches may be combined with biomarkers (e.g., CSF tau and ß-amyloid levels or molecular imaging) to assess how LC neurodegeneration may drive clinical and pathological manifestations of AD. As the AD field moves further toward early disease detection, robust animal models will be essential for understanding early disease mechanisms pertaining to the LC-NE system.

In conclusion, the study by Chalermpalanupap and colleagues provides longitudinal evidence that a combination of LC neurodegeneration and tau pathology synergistically impairs learning and memory and exacerbates neuropathology in a transgenic mouse model of tauopathy. Taken together with known findings from amyloid-based transgenic models, LC degeneration may modulate both tau and ß-amyloid pathology to significantly increase neurodegeneration, neuroinflamation and cognitive impairment. These results lend further support to targeting the LC-NE system as a potential therapeutic strategy for both halting disease progression and ameliorating cognitive deficits in AD.

## Author contributions

MB prepared and wrote the manuscript. AE, DH, and ED wrote and edited the manuscript.

### Conflict of interest statement

The authors declare that the research was conducted in the absence of any commercial or financial relationships that could be construed as a potential conflict of interest.
